# Spermiological injuries of humans and rats in diabetes linked oxidative stress: A curative approach by ex vivo study using aqueous-ethanol extract of Aloe vera (L.)

**DOI:** 10.5935/1518-0557.20250034

**Published:** 2025

**Authors:** Dibya Pal, Kuladip Jana, Sukriti Hazra, Dipanwita Mitra, Debidas Ghosh

**Affiliations:** 1 Molecular Medicine, Nutrigenomics and Public Health Research Laboratory, Department of Bio-Medical Laboratory Science and Management, Vidyasagar University, West Bengal, India; 2 Division of Molecular Medicine, Bose Institute, EN 80, Sector-V, Bidhannagar, Kolkata-700091, India

**Keywords:** diabetes, spermiological disorders, *ex vivo* study, oxidative stress, apoptosis, Aloe vera (L.)

## Abstract

**Objective:**

Among the co-morbidities associated with diabetes, male infertility is rising exponentially. This study highlights the curative effects of the aqueous-ethanol extract of *Aloe vera* (L.) (AEAV) on spermiological injuries in both humans and rats with diabetes, utilizing more sensitive methods for assessing DNA fragmentation and apoptosis for enhanced validation.

**Methods:**

Streptozotocin was used to induce diabetes in Wistar rats. Sperm were collected from the epididymal lumen of both control and diabetic rats. Semen specimens were obtained from normoglycemic and diabetic individuals by masturbation after 4 days of sexual abstinence. Sperm samples were incubated separately in the presence of 10, 20, and 40 mg of AEAV per 10 mL of ex vivo media for 1 or 2 hours, in a doseand duration-specific manner, to evaluate its effect on the spermiological parameters. Toxicity was assessed in the metabolic organs and the prostate of the rats.

**Results:**

Sperm motility, viability, genomic integrity, apoptosis, and DNA fragmentation, assessed by Comet and Annexin-V assays, were significantly impacted in diabetic samples from rats and humans compared to controls, suggesting a potential link to infertility. The extract effectively corrected all the sperm oxidative stress injuries, with the most notable recovery observed at a dose of 20 mg/10 mL. Reduced activities of antioxidant enzymes and elevated levels of lipid oxidation-peroxidation in sperm, testis, and metabolic organs indicate oxidative stress caused by diabetes, which were improved with the extract in a doseand duration-specific manner. Chromatogram analysis, backed by literature and research, primarily revealed the presence of flavonoids and phenolic compounds in the extract.

**Conclusions:**

The AEAV shows promising curative potential for diabetes-associated spermiological injuries.

## INTRODUCTION

Diabetes is now the fourth leading cause of death globally, following cancer, cardiovascular diseases, and lower respiratory tract diseases (Balakumar *et al.*, 2016). This epidemic not only burdens the healthcare system but also significantly impacts the quality of life and overall well-being of individuals. Among the myriad complications associated with diabetes, oxidative stress-induced sperm damage stands out as a critical yet often overlooked consequence, profoundly affecting male reproductive health. According to literature, 35-51% of diabetic males suffer from subfertility or infertility (Lotti & Maggi, 2023). Male infertility in diabetes primarily results from oxidative stress imposed on the testis as well as directly on germ cells (Lotti & Maggi, 2023). The rising prevalence of diabetes in the community makes addressing its adverse impact on male fertility an important public health issue. The mechanisms underlying oxidative stress in diabetes are intricate and involve multiple factors. Hyperglycemia, the hallmark of diabetes, initiates a cascade of metabolic disturbances that trigger the overproduction of reactive oxygen species (ROS). These highly reactive species can cause extensive cellular damage by oxidizing lipids, structural and functional proteins, and nucleic acids (Juan *et al.*, 2021).

In the context of male reproductive health, delicate spermatozoa are particularly vulnerable to oxidative injury due to their rich content of polyunsaturated lipids, acting as prooxidants and limiting antioxidant defenses (Alahmar, 2019). The resulting sperm oxidative stress (SOS) not only impairs sperm motility, morphology, and viability but also compromises DNA integrity through histone incorporation-induced deprotamination, interfering with successful fertilization and embryogenesis, ultimately resulting in infertility (Walke *et al*., 2023). The relationship among diabetes, oxidative damage, and gonadal dysfunction or infertility has been well documented by several epidemiological, preclinical, and clinical studies. Investigations have consistently confirmed that men with diabetes exhibit significantly higher levels of oxidative stress markers in their seminal fluid compared to non-diabetic individu*als* (Amiri *et al*., 2011). Furthermore, diabetes is often associated with reduced sperm quality and altered semen parameters that diminish fertility capability, correlating with increased rates of subfertility and infertility (Sarkar *et al.*, 2019). This interaction between diabetes and reproductive dysfunction necessitates a comprehensive understanding of the underlying mechanisms to develop effective therapeutic strategies. Among several pathways that contribute to heightened oxidative stress in individuals with diabetes, one such pathway involves advanced glycation end products (AGEs), which are formed through the non-enzymatic binding of glucose to proteins. AGEs accumulate in tissues and provoke oxidative stress through receptor interactions. Additionally, mitochondrial dysfunction, a common feature in diabetes, exacerbates ROS production, further perpetuating cellular damage (Zhang *et al*., 2023). The interplay among these pathways creates a vicious cycle that induces oxidative injury, amplifying the detrimental effects on sperm cells and potentially leading to infertility.

Finding the molecular mechanisms underlying diabetes-induced SOS is crucial for identifying potential therapeutic targets. Currently, many anti-diabetic drugs are available that improve blood glucose homeostasis, but they do not provide significant protection against male subfertility or infertility in the diabetic state (Alzain *et al.*, 2021). Antioxidant defense mechanisms are essential for counteracting SOS and restoring fertility in the management of diabetic patients. However, in diabetes, the balance between ROS production and antioxidant capacity often tilts in favor of oxidative stress (Matough *et al.*, 2012). Therapeutic strategies aimed at enhancing endogenous antioxidant defenses or exogenous supplementation with antioxidants have shown promise in reducing oxidative damage and improving sperm function in individuals with diabetes; however, such chemically synthetic antioxidants may have carcinogenic and hepatotoxic effects (Lourenço *et al*., 2019). From that perspective, there is need for global search of agents from natural resources with minimal or no toxic effects that can effectively address diabetic carbohydrate metabolic disturbances and associated male reproductive comorbidities, with a focus on achieving one-drug multitarget therapy as per the guidance of WHO (Sarkar *et al*., 2019).

Moreover, continuing from our previous reports in this line (Pal *et al.*, 2024a; 2024b), the present study was designed to further investigate the assessment of DNA fragmentation by incorporating more sensitive and precise methods, such as Annexin-V and the Comet assay, over terminal deoxynucleotidyl transferase dUTP nick-end labeling (TUNEL), along with the chromatin packaging status of sperm assessed by Acridine orange. We also included the assessment of prostatic inflammation status through evaluation of acid phosphatase activity, an important indicator of sperm activity, adding another dimension to our previous observations in this line (Pal *et al*., 2024b). These studies provide clear, valid, and early insights into diabetes-induced sperm damage and its potential recovery through *Aloe vera (L.).* Additionally, high-performance liquid chromatography (HPLC) was employed here to identify the most abundant phytomolecule(s) in the extract, which is more effective for extract analysis compared to other chromatographic techniques, including liquid chromatography mass spectrometry (LC-MS) (Steinmann & Ganzera, 2011; Gupta *et al.*, 2022), used in our previous study (Pal *et al.*, 2024b). Furthermore, since the study has implications for sperm processing in assisted reproduction by enhancing sperm quality for successful pregnancy outcomes, early detection of sperm apoptosis and DNA fragmentation is very helpful in differentiating semen samples from the perspective of their eligibility for this purpose.

Currently, plant resources are regarded as among the most important bio-resources for drug development (Nasim *et al*., 2022). Numerous herbs have demonstrated promising antidiabetic activity, including *Momordica charantia, Allium cepa* (also known as Allium), and *Gymnema* sylvestre. *Aloe vera (L.)* is an example of such herbs that possess antidiabetic and antioxidative activities (Tran *et al.*, 2020). The literature highlights some contradictory results regarding this herb’s effects on spermiological sensors and oxidative damage (Olugbenga *et al.*, 2011; Pal *et al.*, 2024a; 2024b). The phytomolecule(s) found in *Aloe vera (L.)* extracts exhibit dual effects, where a very low dose acts as a prooxidant, while a moderate dose demonstrates antioxidant activities, which may be attributed to variations in dose and treatment dura*t*ion (Olugbenga *et al*., 2011; Behmanesh *et al*., 2018; Akinola *et al*., 2021; Pal *et al*., 2024a). Against this backdrop, the current *ex vivo* study aims to resolve the existing contradictions concerning the doses of *Aloe vera (L.)* that are non-toxic and show optimal therapeutic effects for correcting sperm injuries and oxidative damage, with a primary focus on male infertility management in diabetes, one of its major co-morbidities.

This study provides a blueprint for future research aimed at developing a herbal drug for sperm processing and optimization, ultimately enhancing fertility outcomes in Artificial Reproductive Technology (ART). The duration of this *ex vivo* study does not permit genomic modulation or the related alterations in cellular activity.

## MATERIALS AND METHODS

### Plant collection

The matured leaves (two to three years old) of *Aloe vera (L.)* were collected once during the monsoon season from a nearby nursery of Vidyasagar University in Midnapore, West Bengal, India. Care was taken to select healthy, undamaged leaves with a thick, fleshy texture, as these indicate high mucilage and bioactive content. The leaves were authenticated (herbarium no. *Aloe vera (L.)/*VU/Bio/06/19) by the taxonomist from the Botany and Forestry Department at Vidyasagar University, Midnapore, Paschim Midnapore, West Bengal, India.

### Extraction of *Aloe vera (L.)* using an aqueous-ethanol solvent

The mucilaginous gel sandwiched between the outer green rinds of *Aloe vera (L.)* was collected. Deionized water (DIW) and ethanol were mixed in a ratio of 2:3. Five hundred milliliters of the gel were added to 1 liter of the above aqueous-ethanol mixture, which was kept under normal atmospheric conditions for 2 days and then concentrated by rotary evaporation (EYELA N-1200A, Japan). The concentrated aqueous-ethanol extract of *Aloe vera (L.)* (AEAV) was lyophilized and stored in a refrigerator for experimental use (Pal *et al*., 2024a).

### Ethical approval

The experimental design involving both rats and humans was approved by the Institutional Committee for Ethical Clearance, with the sanctioned reference numbers VU/IAEC-I/DG-1/3-15/19 and VU/IHEC-5/15-24, respectively. The protocol for conducting the experimentation, as well as the standard norms for the onset of diabetes in animals, was sanctioned by the Committee for Control and Supervision of Experiments on Animals (CCSEA), Government of India. Completed consent forms from human participants were collected prior to the collection of semen samples. The collection and transportation of semen samples to the laboratory were carried out with the assistance of paramedical staff, following the standard and routine protocol of the WHO (2010).

### Selection criteria of subjects

#### (i) For rats

Streptozotocin-injected model diabetic rats with fasting blood glucose (FBG) levels of 200-250 mg/dL were selected for this study. Out of the twenty model diabetic rats, sixteen met these criteria and were deemed eligible for inclusion in the diabetic group. The remaining sixteen normoglycemic rats, with FBG levels ranging from 75-85 mg/dL, were assigned to the control group.

#### (ii) For humans

Semen samples were collected from a control group of sixteen healthy, normoglycemic young adults, aged 25-30 years (FBG 75-85 mg/dL). These individuals met the spermiological parameters within the fertile range according to WHO reference values and did not have major complications such as hypertension, infections in the reproductive or urinary tracts, drug or smoke addiction, or thyroid pathophysiology. They were selected from the community after proper information collection and were supervised and monitored under the direct guidance of a physician from the medical unit at Vidyasagar University, Midnapore. Another group of sixteen individuals, matched in age and dietary status to the control group, was included in the diabetic group, with FBG levels ranging from 200-250 mg/dL for the past two years and glycated hemoglobin (HbA1c) levels exceeding 6.5%, without other major co-morbidities. Samples were collected from the ‘National Urban Health Mission’ center in Midnapore town, under the direct guidance of the relevant doctor and paramedical staff at that center.

### Experimental framework

#### (i) For rats

Thirty-six healthy, normoglycemic, fertile male Wistar rats (*Rattus norvegicus*), aged two and a half months and weighing 130±10 g, were acquired from a CCSEA-certified supplier. Before initiating the experiment, the animals acclimatized for ten days in a controlled environment with a room temperature of 25±2°C, regulated humidity, and a 1:1 light/dark cycle over a 24-hour duration. They had access to water and food *ad libitum*. Afterward, experimental diabetes was induced in twenty rats using a single injection of streptozotocin (STZ) in the leg muscle at a dose of 40 mg/kg of body weight (Pal *et al*., 2024a). To validate the sustained diabetic state of the STZ-injected rats, fasting blood glucose (FBG) levels were monitored at two-day intervals for ten days, with FBG levels between 200-250 mg/dL used as the criteria for conducting the study. Animals meeting these criteria were housed in a controlled facility for 28 days, with intermittent checks of FBG levels to confirm long-standing diabetes. The remaining sixteen rats, which did not receive the STZ injection, were kept under the same laboratory conditions for 28 days and served as the control group. For tissue and biological sample collection, the rats were euthanized by cervical dislocation following CCSEA ethical standards, supervised by a veterinarian to minimize strain on the organs. The cauda epididymis of each rat was carefully dissected and immediately immersed in 1000 µL of pre-warmed isotonic saline solution (0.9% NaCl) to maintain physiological conditions and prevent osmotic stress on the stored sperm cells. Gentle mincing of the tissue allowed the spermatozoa to disperse into the solution, forming a suspension suitable for subsequent *ex vivo* analysis. The liver, kidney, testis, and prostate were collected from the rats, with surface blood and tissue fluid cleaned and appropriately stored for biochemical analysis in this study.

#### (ii) For humans

Semen was collected from sixteen diabetic and sixteen normoglycemic subjects according to the standard laboratory guidelines of the WHO. After four days of abstinence from sexual activity, semen sample collection was performed through masturbation into a clean, sterile, polystyrene container in a private room to ensure the sample’s integrity and privacy. The total ejaculate was collected and maintained at 37℃ for its natural coagulation and reliquefaction, which was completed within 1 hour. After incubation, the samples were considered for the *ex vivo* study (WHO, 2010; Pal *et al*., 2024b).

### Randomisation and probability sampling

Participants from both groups were chosen using a simple randomization method that adhered to the specified inclusion and exclusion criteria. The influence of confounding factors was minimized in both rat and human samples through routine surveillance and monitoring to examine the effects of independent variables on dependent variables in a cause-and-effect relationship study. This eliminated personal choice, bias, and preference regarding subject inclusion in the study.

### Outcomes and single-blind study experimentation

Four major outcomes were measured: i) diabetes-linked DNA breakage and apoptosis of sperm, along with their recovery using *Aloe vera (L.)* extract and employing the most sensitive and valid sensors, ii) the deterioration of sperm fertility sensors in diabetes and their correction through the aforementioned extract, iii) oxidative stress injuries in sperm during diabetes and their recovery due to *Aloe vera (L.)* extract in a doseand duration-dependent manner, and iv) for examining sperm injuries in diabetes and their recovery by the extract, we adopted Comet, Annixin V, and Acridine orange assays, which are more specific and accurate sensors with a high rate of true positive results. This approach builds on our previous study using TUNEL and nuclear chromatin decondensation (NCD) of sperm, common sensors in this context, which have lower sensitivity and a higher rate of false positive outcomes. To assess the quality of these outcomes, we implemented a single-blind study model to minimize bias and partiality during sample collection and processing. This ensures that the researchers involved in sample handling were unaware of the groups, sub-groups, and sub-sub groups as per the study design. This approach also reduces experimental error and enhances the scientific merit, validity, accuracy, and acceptability of the study.

#### *Ex vivo* media preparation

Krebs-Ringer Bicarbonate (KRB) solution with a pH of 7.4 was used as an *ex vivo* medium. The ingredients, at the standard strength, were mixed with 0.8 liters of DIW. The volume was adjusted to 1 liter in a volumetric flask using DIW (Pal *et al*., 2024b).

#### *Ex vivo* study design

Human semen samples, along with rat epididymal washed sperm samples, were double diluted by mixing with KRB isotonic solution. The samples of human and rat sperm were divided into the following groups: control, AEAV-uncharged diabetes, and AEAV-charged diabetes. Based on the duration of the sustained charging period, each of these groups was subdivided into two sub-groups - 1 hour of charging and 2 hours of charging. Only the AEAV-charged 1 and 2 hours sub-groups were further categorized into subsub-groups according to the dose of AEAV charging: 10 mg AEAV-charged diabetes (1 mg/mL), 20 mg AEAV-charged diabetes (2 mg/mL), and 40 mg AEAV-charged diabetes (4 mg/mL). The categories of the above grouping, sub-grouping, and sub-sub-grouping were as follows:

Control group: Sixteen test tubes were assigned, with each containing 10 mL of KRB solution mixed with 0.5 mL of sperm suspension from either a normoglycemic, fertile rat or 0.5 mL of semen from a normoglycemic individual. This was followed by supplying a gas mixture, as stated later, at a fixed rate of 30 bubbles per minute and an *ex vivo* duration of experimentation mentioned earlier. Based on the *ex vivo* duration of 1 or 2 hours, the group was divided into two sub-groups for rats and another two sub-groups for humans. An additional 16 test tubes were used for each type of tissue slice, i.e., liver, kidney, testis, and prostate, for the control group of rats only, which were incubated for 2 hours.

AEAV-uncharged diabetes group: This group was categorized into two subgroups based on the duration of the experiment. Each test tube contains 10 mL of KRB solution and 0.5 mL of epididymal washed sperm suspension from diabetic rats or 0.5 mL of semen from diabetic individuals. The solution was supplied at the same rate as the control for 1 or 2 hours. The duration-dependent two subgroups for rats and the other two subgroups for humans were designated as uncharged diabetes sub-groups (UDSGs). The extract was not added to the *ex vivo* media. Like the control subgroups, 16 test tubes were used for each type of tissue sample mentioned above.

10 mg AEAV/10 mL of ex vivo media-charged diabetes sub-sub-group: In the 10 mL solution of KRB, 0.5 mL of diabetic rat sperm suspension or 0.5 mL of diabetic person’s semen was added along with 10 mg AEAV (1 mg/mL) and processed as previously for 1 or 2 hours. Subgroups for rats and humans were allocated as previously outlined in UDSGs. Based on the 10 mg/10 mL dose for 1 and 2 hours, the subgroups were divided into four sub-subgroups. Additionally, 16 more test tubes were assigned for each of the aforementioned tissue slices from the diabetes group of rats, which underwent the same incubation duration, i.e., 2 hours.

20 mg AEAV/10 mL of ex vivo media-charged diabetes sub-sub-group: Diabetic rat sperm suspension of 0.5 mL or diabetic person’s semen volume of 0.5 mL mixed in 10 mL of KRB solution. The *ex vivo* preparation was allowed to charge with 20 mg AEAV (2 mg/mL) and processed as previously described for 1 or 2 hours. Subgrouping and sub-subgrouping of rat and human samples were performed as previously described for sub-subgroups. Another 16 test tubes were assigned for each type of the above tissues from diabetic rats, which were exposed for 2 hours with 20 mg/10 mL of AEAV.

40 mg AEAV per 10 mL of ex vivo media-charged diabetes sub-sub-group: An isotonic KRB solution of 10 mL was mixed with 0.5 mL of diabetic rat sperm suspension or 0.5 mL of diabetic person’s semen along with 40 mg AEAV (4 mg/mL). The sample mixture underwent incubation for 1 or 2 hours. Subgrouping and sub-sub-grouping of rat and human samples were conducted as per previous design. Additionally, 16 test tubes were used for each mentioned tissue of diabetic rats, exposed for 2 hours to 40 mg per 10 mL of AEAV.

Sixteen test tubes were designated for each control, AEAV-unexposed diabetes, as well as for durationand dose-specific AEAV-charged diabetes sub-sub-groups. In total, 640 test tubes for 640 samples encompassing all sub-groups and sub-sub-groups were included in this study. Each sample was placed in an incubator set to 37℃ for 1 to 2 hours, exposed to a steady stream of a gas blend containing O_2_ and CO_2_ at a 9.5:0.5 ratio, while maintaining the specified flow rate of 30 bubbles per minute. Following the exposure of sperm cells to the *ex vivo* solution for 1 or 2 hours, spermiological analysis was conducted immediately. The sperm pellet, along with all the aforementioned tissue samples, was subsequently processed to assess peroxidase and glutathione-S-transferase activities and to quantify conjugated diene levels after the 2-hour incubation (Pal *et al*., 2024b). There was no option for long-term sperm/semen sample storage, as sperm analysis was performed immediately. For the time being, samples were stored in the incubator at 37℃.

### Motile and viable sperm percentage

The percentage of forward-progressive spermatozoa was counted from a 20-µL *ex vivo* solution of groups, subgroups, and sub-subgroups. Eosin-unstained spermatozoa were considered viable and expressed as a percentage (WHO, 2010).

### Genomic integrity

A solution containing sperm samples from *ex vivo* media was used to prepare a smear on a glass slide, which was then air-dried. After fixation in Carnoy’s fixative, the slides were stained with 0.1% Acridine orange in a sodium phosphate buffer of citric acid (pH 7.4). The stained slides were washed in DIW twice and subjected to fluorescence microscopic observation (Zeiss, Germany). Orange-colored sperm indicate the intercalation of Acridine orange at double-stranded DNA break points and highlight DNA disintegrity (Ansari *et al*., 2018).

### Annexin V assay

Sperm with fragmented DNA were analyzed by staining with propidium iodide (PI) and fluorescein isothiocyanate (FITC)-labeled annexin V (FLAV) using a flow cytometer (FACSCalibur, Beckton, Dickinson, San Diego, CA, USA). Ten thousand spermatozoa were counted for each sample. Channel FL1 was used to measure FLAV-positive cells, while Channel FL2 measured PI-tagged sperm cells (Oosterhuis *et al*., 2000).

### Comet assay

Glass slides were evenly pre-coated with agarose and allowed to solidify at 4℃. Sperm cells present in *ex vivo* media were mixed with low-melt agarose and placed on an agarose pre-coated slide, which was further allowed to solidify at the same temperature. The prepared slides were immersed in lysis buffer for 1 hour at 4℃ in the dark, rinsed with DI water, and subsequently transferred to electrophoresis buffer for 30 minutes. The processed slides were immersed three times in a neutralized buffer for 5 minutes and stained with ethidium bromide. Sperm cells with comet tails were viewed through a fluorescence microscope, counted, and the results were expressed as a percentage of the total count (Martínez-Luna *et al*., 2015).

### Oxidative stress markers of metabolic and reproductive organs

An ice-cold phosphate-based buffer solution (pH 7.4) was prepared to homogenize targeted tissues at a concentration of 50 mg/mL. The supernatant was collected after centrifugation. Peroxidase, glutathione-S-transferase (GST) enzyme activities, and conjugated diene levels in the liver, kidney, and testis of rats were measured following standard biochemical procedures after treating the organs with AEAV at the specified doses in *ex vivo* KRB (pH 7.4) media. This was followed by the supply of a gas mixture consisting of 95% CO_2_ and 5% O_2_ for a duration of 2 hours at 37℃ (Sadasivam & Manickam, 1996; Habig *et al*., 1974; Slater, 1984), along with controls and UDSGs.

### Toxicity markers of metabolic and reproductive organs

Acid and alkaline phosphatase activities in the prostate, along with aspartate aminotransferase (AST) and alanine aminotransferase (ALT) activities in the kidneys and liver of rats, were measured using biochemical methods. The tissue slices in *ex vivo* KRB (pH 7.4) media were treated with AEAV extract in a dose-specific manner, followed by the administration of a gas mixture as previously describ*e*d for 2 hours at 37℃ (Malamy & Horecker, 1966; Mattei *et al*., 1985; Shree *et al*., 2017; Jagadeesan & Kavitha, 2006), along with the control and UDSGs.

### DPPH scavenging activity assessment

The methanolic solution of the AEAV was prepared at different concentration ranges (5 - 100 mg/lit). In a separate test tube, a DPPH solution (100 µM) in methanol was also prepared. The DPPH solution and plant extract were mixed in a 2:1 ratio and kept away from light for half an hour. The optical density was measured at 517 nm (Rahman *et al*., 2015).

### Phytomolecule(s) analysis

The phytomolecule(s) present in AEAV were analyzed qualitatively through biochemical tests (Ara *et al.*, 2019).

### Total flavonoid and phenolic contents of AEAV

A colorimetric process was employed to estimate the total flavonoid and phenolic content in the plant extract using AlCl_3_ and Folin-Ciocalteu reagents, respectively. Both flavonoid and phenolic contents were calculated using the standard graphs of quercetin and gallic acid with a spectrophotometer (Thermo Scientific, USA) (Bera *et al*., 2014).

### High Performance Liquid Chromatography (HPLC)

The plant extract was dissolved in deuterium oxide. The filtrate was collected using a filter with a 0.45 μm pore size and transferred to HPLC sample vials. The mobile phase was prepared with water (0.1% formic acid) and acetonitrile (0.1% formic acid). For HPLC analysis of AEAV, a C18 column was used. Twenty μL of the extract was injected, and the graded elution was initiated with 95% water and 5% acetonitrile, elevated to 95% acetonitrile over 30 minutes, maintained for 10 minutes, then returned to starting conditions within 5 minutes, followed by re-equilibration for 10 minutes. The phytomolecules were detected at a wavelength of 254 nm. To identify compounds, chromatogram fingerprinting was performed using a chromatogram database (Nguyen *et al*., 2018; Nwaso-Sunday *et al*., 2024).

### Data interpretation

Analysis of variance (ANOVA) followed by post-hoc analysis was conducted to assess the impact of the controlled variable on the uncontrolled variables. The probability level of *p*<0.05 was regarded as the minimum threshold for significant cause-and-effect relationship studies focusing on the variables (Sokal & Rohle, 1995).

### Quality control of data

To enhance the acceptability, accuracy, and precision of the observational output while minimizing discrepancies in the experiment and strengthening quality assurance in this *ex vivo* study, the sample size was increased in each sub-group and sub-sub-group, i.e., n=16, to reduce type I and type II errors in the significance analysis using statistical tools.

## RESULTS

### Motile sperm

A notable decrease (*p*˂0.05) in the forward progressive motile sperm (FPMS) percentage was observed in the 0 hour value of the diabetes group compared to the time-matched control in both humans and rats. After 1 and 2 hours of incubation, the percentage of FPMS declined in both the control and UDSGs relative to the 0 hour value. The level of this sensor at 1 and 2 hours in the uncharged diabetic group was lower compared to the control group. A significant (*p*˂0.05) duration-dependent decrease in FPMS was noted in the AEAV uncharged diabetic group. However, treating the sperm with the extract significantly reversed the downward trend in FPMS counts after both 1 and 2 hours of incubation. There was no significant decrease in FPMS even after 2 hours of treatment compared to 1 hour of treatment, highlighting the extract’s recovery capacity. Dose-dependent improvement in this parameter was not observed significantly (*p*˂0.05) when the treatment duration remained fixed. This trend was consistent in both humans and rats (Table 1).

**Table 1:** Effect of AEAV at different concentrations and durations on the sperm cells of diabetic human and rat sample in *ex vivo* study.

Parameters	Rat											
	0 hour		1 hour of incubation					2 hours of incubation				
	Control group	AEAV uncharged diabetes	Control group	AEAV uncharged diabetes	10 mg AEAV charged diabetes	20 mg AEAV charged diabetes	40 mg AEAV charged diabetes	Control group	AEAV uncharged diabetes	10 mg AEAV charged diabetes	20 mg AEAV charged diabetes	40 mg AEAV charged diabetes
% of FPMS	52.36±0.78	33.27±0.51^b^	48.14±0.96^c^	23.2±0.67^d^	30.25±0.58^e^	30.73±0.47^e^	31.03±0.61^e^	44.93±0.64^f^	16.69±0.56^g^	29.02±0.30^e^	29.23±0.41^e^	29.70±0.55^e^
% of viable sperm	87.28±0.62^a^	45.68±0.71^b^	83.41±0.55^c^	34.50±0.58^d^	41.44±0.66^e^	42.23±0.67^e^	42.65±1.09^e^	79.05±0.37^f^	28.75±0.50^g^	41.47±0.3^e^	40.77±0.47^e^	41.96±0.38^e^
Genomic integrity	76.19±2.65^a^	43.25±1.18^b^	71.15±1.69^c^	32.15±1.24^d^	39.16±1.26^e^	39.68±1.37^e^	40.06±1.64^e^	66.16±2.33^f^	21.16±1.65^g^	38.11±1.02^e^	39.16±1.09^e^	39.84±1.22^e^
Parameters	Human											
	**0 hour**		**1 hour of incubation**					**2 hours of incubation**				
	Control group	AEAV uncharged diabetes	Control group	AEAV uncharged diabetes	10 mg AEAV charged diabetes	20 mg AEAV charged diabetes	40 mg AEAV charged diabetes	Control group	AEAV uncharged diabetes	10 mg AEAV charged diabetes	20 mg AEAV charged diabetes	40 mg AEAV charged diabetes
% of FPMS	59.41±1.85^a^	31.16±2.34^b^	54.39±1.45^c^	22.91±1.26^d^	29.09±0.50^e^	29.19±0.56^e^	29.35±0.63^e^	51.34±1.06^f^	13.03±0.84^g^	27.69±0.79^e^	27.89±0.57^e^	28.19±0.54^e^
% of viable sperm	85.91±1.91^a^	49.33±0.71^b^	81.16±1.27^c^	40.96±0.83^d^	45.23±0.39^e^	46.11±0.37^e^	47.55±0.89^e^	78.55±0.54^f^	33.78±1.33^g^	44.68±1.43^e^	45.35±0.68^e^	46.31±0.58^e^
Genomic integrity	71.25±1.24^a^	41.62±1.21^b^	67.15±1.25^c^	30.20±1.39^d^	36.16±1.26^e^	36.94±1.35^e^	37.11±1.21^e^	63.36±1.38^f^	20.25±1.68^g^	35.28±1.57^e^	36.61±1.25^e^	37.31±1.36^e^

Human and rat samples in an ex vivo study. Data represented mean value±SEM, n=16. Mean with different superscripts in every row for rat or human spermiological data vary from one another at significant level, *p*<0.05. ANOVA followed by posthoc analysis were used as statistical tools and for the significance study.

### Viable sperm count

Incubation of human and rat sperm samples for durations of 1 or 2 hours showed a progressive decrease in the percentage of viable sperm in UDSGs. In contrast, a significant improvement in live sperm was noted after 1 or 2 hours of incubation with 10, 20, and 40 mg AEAV. Statistically nonsignificant (*p*>0.05) dose-dependent improvement was observed among the subgroups receiving 10, 20, and 40 mg AEAV, considering the fixed duration of charging (Table 1).

### Genomic integrity

The green fluorescence of sperm indicated the presence of double-stranded DNA, and based on this assessment, genomic integrity was evaluated under various conditions. The results at 0 hour showed a significantly lower percentage of sperm emitting green fluorescence (SEGF) in the diabetes samples of humans and rats compared to the respective control groups. After 1 and 2 hours of incubation, the UDSGs exhibited a significant reduction (*p*<0.05) in SEGF compared to the 0-hour value of the same group. Interestingly, the number of SEGF was higher in the 10, 20, and 40 mg AEAV groups after 1 and 2 hours of charging than in the UDSGs from the 1 and 2-hour incubation periods, respectively. However, a statistically non-significant (*p*>0.05) variation was observed in the percentage of SEGF after 1 and 2 hours of incubation when comparisons were made within the 10, 20, and 40 mg AEAV dose-charged diabetes conditions in both humans and rats (Table 1).

### Annexin V assay

**Figure 1:**
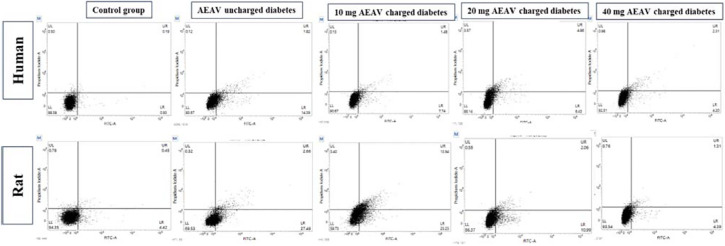
DNA fragmentation assay of human and rat sperm cells using Annexin-V in a diabetic state and its rectification by AEAV after 2 hours of exposure.

Annexin V can selectively bind to membrane phosphatidylserine in cells with compromised membrane phospholipid asymmetry, a characteristic of apoptotic cells with DNA fragmentation. The percentage of fragmented DNA in human sperm after 2 hours of exposure was 0.93%, 14.33%, 7.74%, 6.43%, and 4.20% in the control, diabetes, and 10, 20, and 40 mg AEAV-charged diabetes sub-groups, respectively. In the case of rats, the corresponding percentages were 4.42%, 27.49%, 23.23%, 10.99%, and 4.39% in the control, diabetes, and 10, 20, and 40 mg AEAV-charged sub-groups, respectively (Figure 1).

### Comet assay

**Figure 2:**
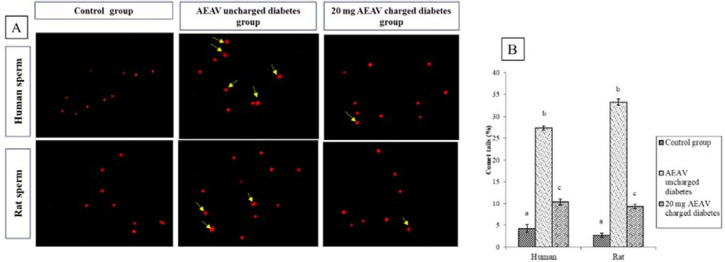
Comet assay of human and rat sperm cells. (A) Representation of the microscopic view of sperm with Comet tails, (B) percentage of sperm with Comet tails in control, diabetes, and 20 mg AEAV charged conditions highlighting the curative effect of AEAV. Bars with different superscripts vary from one another at a significant level, *p*<0.05 (n=16). ANOVA followed by post-hoc analysis was used as the statistical tools for the significance study.

The comet assay was conducted on the 20 mg charged AEAV sub-group after 2 hours of charging, compared to the control group in both humans and rats, because this dose produces the optimal level of rectifications in most of the sensors among the tested doses. The percentage of sperm with a comet tail increased in the UDSGs compared to the control. Significant improvement was noted after charging the diabetic sample for 2 hours with 20 mg of AEAV, which is the effective threshold dose for optimal activity (Figure 2).

### Oxidative stress marker

Charging slices of the liver, kidney, and testis *ex vivo* with doses of 10, 20, and 40 mg of AEAV for 2 hours resulted in significant remediation of antioxidant enzyme activities and conjugated diene levels compared to the UDSGs. No significant (*p*>0.05) dose-dependent improvement was observed in these markers within the specified duration (Table 2).

**Table 2:** Curative effect of AEAV on oxidative stress and toxicity markers in diabetic rats after 2 hours of exposure to AEAV in an *ex vivo* condition.

Parameters	Control group	AEAV uncharged diabetes	10 mg AEAV charged diabetes	20 mg AEAV charged diabetes	40 mg AEAV charged diabetes
**Peroxidase activity in liver (unit/mg of tissue)**	6.75±1.26^a^	2.36±0.42^b^	3.36±0.96^c^	3.45±0.63^c^	3.57±0.54^c^
**Peroxidase activity in kidney (unit/mg of tissue)**	5.68±0.68^a^	1.93±0.36^b^	2.83±0.32^c^	2.91±0.65^c^	2.99±0.67^c^
**Peroxidase activity in testis (unit/mg of tissue)**	4.63±0.94^a^	1.06±0.45^b^	2.21±0.63^c^	2.25±0.36^c^	2.26±0.68^c^
**GST activity in liver (µM/min/mg of tissue)**	0.751±0.047^a^	0.233±0.039^b^	0.301±0.021^c^	0.306±0.046^c^	0.311±0.034^c^
**GST activity in kidney (µM/min/mg of tissue)**	0.536±0.061^a^	0.173±0.041^b^	0.294±0.023^c^	0.301±0.047^c^	0.309±0.37^c^
**GST activity in testis (µM/min/mg of tissue)**	0.456±0.032^a^	0.102±0.026^b^	0.171±0.051^c^	0.176±0.054^c^	0.183±0.061^c^
**CD level in liver (unit/mg of tissue)**	264.03±3.54^a^	467.31±4.45^b^	433.12±4.11^c^	428.26±4.37^c^	426.61±3.64^c^
**CD level in kidney (unit/mg of tissue)**	197.49±3.64^a^	378.36±4.21^b^	357.32±4.26^c^	353.65±4.31^c^	349.30±4.95^c^
**CD level in testis (unit/mg of tissue)**	159.36±4.01^a^	329.36±4.34^b^	301.25±4.65^c^	296.26±4.63^c^	293.33±3.44^c^
**Acid phosphatase activity in prostate (mg/PNP/mg of tissue/h)**	15.59±2.23^a^	34.23±1.47^b^	30.03±1.24^c^	29.66±1.14^c^	29.14±1.09^c^
**Alkaline phosphatase activity in prostate (mg/PNP/mg of tissue/h)**	12.36±1.19^a^	31.25 ±1.32^b^	27.21 ±1.10^c^	26.68 ±1.53^c^	26.24±1.38^c^
**AST activity in liver (unit/mg of tissue)**	22.32±1.25^a^	37.15±1.05^b^	36.14±1.36^b^	35.60±1.67^b^	35.15±1.49^b^
**AST activity in kidney(unit/mg of tissue)**	16.35±2.71^a^	34.64±2.37^b^	33.21±1.25^b^	32.98±1.54^b^	32.45±1.23^b^
**ALT activity in liver (unit/mg of tissue)**	26.16±3.21^a^	41.23±3.28^b^	39.10±2.50^b^	38.56±1.65^b^	37.15±1.35^b^
**ALT activity in kidney (unit/mg of tissue)**	21.32±2.54^a^	39.21±2.35^b^	38.10±1.78^b^	37.66±1.11^b^	37.23±1.54^b^

Data represented mean value±SEM, n=16. Mean with different superscripts in each row vary from one another at significant level, *p*˂0.05. ANOVA followed by post-hoc analysis were used as statistical tools for the significance study.

### Toxicity markers

Recovery in the elevated activities of prostatic acid and alkaline phosphatase was observed in a dose-dependent manner after AEAV charging against UDSGs. However, no significant (*p*>0.05) improvement was observed in hepatic and renal ALT and AST activities in AEAV-charged diabetes groups compared to UDSGs (Table 2).

### DPPH scavenging activity

**Figure 3:**
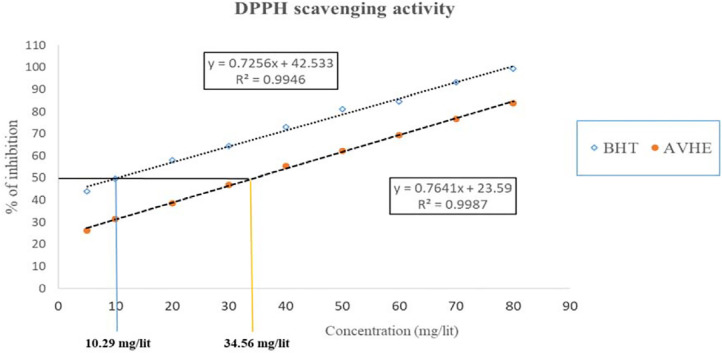
DPPH scavenging activity of AEAV and BHT, where the IC50 values for free radical destruction were 34.56 mg/liter and 10.29 mg/liter, respectively.

Scavenging and inhibition of DPPH by AEAV were observed at different doses. The IC50 values for DPPH scavenging by AEAV and butylated hydroxytoluene (BHT) were 34.56 mg/lit and 10.29 mg/lit, respectively, highlighting its strong antioxidant properties through the free radical scavenging pathway (Figure 3).

### Qualitative screening of phytocompound (s)

For the qualitative analysis of AEAV, the main phytomolecules were alkaloids, phenols, flavonoids, and saponins (Table 3).

**Table 3:** Qualitative and quantitative analysis of the phytocompounds present in AEAV.

Nature of phytocompounds	Qualitative/quantitative screening of AEAV
Flavonoid	+
Alkaloid	+
Phenol	+
Saponin	+
Terpenoid	-
Steroid	-
Glycosides	-
Cardioglycosides	-
Tannins	-
Anthroquinone	-
Flavonoid Content (mg QAE/g)	223.20
Phenolic content (mg GA/g)	223.20

Note: Analysis “(+)” means the presence of phytocompound (s) and “(-)” means the absence of the phytocompound (s) in the extract. QAE: Quercetin; GA: Gallic acid.

### Total flavonoid and phenolic contents of AEAV

The results showed a total flavonoid content of 223.20 mg QAE equivalent per gram of AEAV, while the total phenolic content was 97.34 mg GA equivalent per gram of AEAV (Table 3).

### HPLC

**Figure 4:**
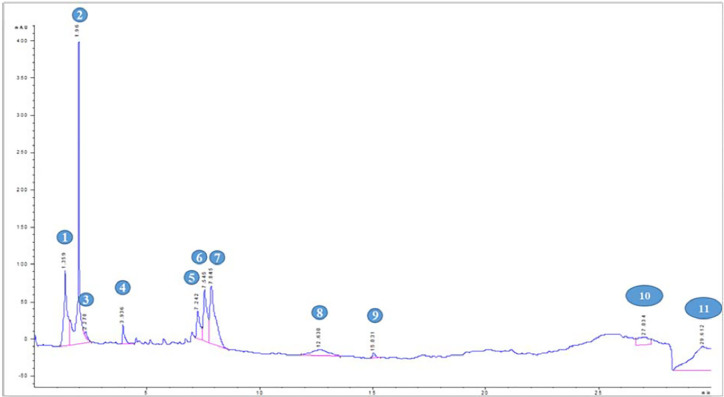
Detection of tentative phytomolecules present in AEAV by HPLC using chromatogram literature or library. The details of each peak (1-11) are described in Table 4.

Out of the 11 peaks, seven peaks, including resveratrol, epi-gallocatechin, 2,4-dihydroxybenzoic acid, methyl gallate, caffeic acid, ferulic acid, and quercetin, were identified and tentatively characterized according to the retention time of the compounds. The above seven phytomolecules belong to the phenolic and flavonoid groups, which were validated with acceptable linearity, repeatability, stability, and recovery of the extract through several runs of the column, presenting phytomolecules as compared to the chromatogram library and literature (Table 4, Figure 4).

**Table 4:** Tentative phytocompounds detected in HPLC analysis of AEAV from comparison with chromatogram library.

Serial no of peaks	Retention time (min)	Name of the phytocompound	Area percentage	Nature
**Peak 1**	1.359	Not detected	12.353	Not detected
**Peak 2**	1.961	Not detected	26.012	Not detected
**Peak 3**	2.278	Not detected	0.569	Not detected
**Peak 4**	3.936	Not detected	2.209	Not detected
**Peak 5**	7.242	Resveratrol	4.177	Polyphenol
**Peak 6**	7.545	Epi-gallocatechin	8.461	Polyphenol
**Peak 7**	7.845	2,4-dihydroxybenzoic acid	14.963	Flavonoid
**Peak 8**	12.63	Methyl gallate	4.823	Phenolic compound
**Peak 9**	15.031	Caffeic acid	0.637	Phenolic acid
**Peak 10**	27.034	Ferulic acid	4.266	Phenolic acid
**Peak 11**	29.612	Quercetin	21.529	Flavonoid

## DISCUSSION

The previous article (Pal *et al.*, 2024a) was a pilot study aimed at selecting the potent extract of *Aloe vera (L.)* to rectify diabetes-induced testicular hypo-function while considering some fundamental sensors in the relevant domains. Following that, another study (Pal *et al.*, 2024b) was designed to detect late-stage apoptosis using the TUNEL assay, which cannot differentiate between apoptotic and necrotic cells and may yield false positives (Kylarová *et al*., 2002). The promising outcomes of the above two studies drive us to extend our observations in-depth to validate the results in this field at a more accurate and highly acceptable level by selecting methods with high sensitivity, specificity, and accurate positive results, especially for SOS-induced DNA fragmentation and sperm apoptosis, to enhance the application of this extract as a sperm charger and enhancer during semen processing in ART. This study overcomes previous limitations by incorporating Annexin-V, Comet, and Acridine orange assays to assess early apoptosis, DNA integrity, and chromatin packaging. The alkaline Comet assay, which is superior to TUNEL, detects both single and double-strand DNA breaks and provides a comprehensive distribution of DNA-damaged sperm (Simon *et al.*, 2014). Annexin-V identifies phosphatidylserine translocation, an early apoptotic marker linked to sperm motility, an aspect where TUNEL cannot provide assessment (Oosterhuis *et al*., 2000). Acridine orange can detect protamine deficiency, a crucial factor affecting sperm DNA stability and maturation, particularly in ART (Hoshi *et al.*, 1996). Furthermore, excessive activities of prostatic phosphatases, associated with premature membrane degradation of sperm and infertility (Verze *et al.*, 2016; Alkhalaf *et al.*, 2020), were evaluated, considering their link to prostate inflammation and diabetes-induced benign prostatic hyperplasia (Dokuyucu *et al.*, 2016; Adaramoye *et al*., 2019).

Diabetes-associated ROS generation in the testicle activates the polyol pathway, where aldose reductase converts glucose to sorbitol, consuming NADPH and reducing glutathione levels, thereby weakening the antioxidant defense system (Tang *et al*., 2019). This metabolic shift also elevates diacylglycerol levels, activating isoforms of protein kinase C, which further stimulate NADPH oxidase to produce more ROS (Jubaidi *et al.*, 2022). Additionally, high glucose levels enhance the formation of AGEs, which bind to the receptor for advanced glycation end products on cell surfaces, triggering signaling cascades that include NF-κB activation, resulting in elevated levels of pro-inflammatory cytokines and further increasing ROS production (Khalid *et al.*, 2022). Moreover, mitochondrial dysfunction in diabetes impairs the efficiency of the electron transport chain (ETC), particularly at complex III, causing electron leakage that increases superoxide formation and lipid peroxidation, compromising the sperm membrane’s structural integrity, leading to the loss of transmembrane potential and impairing the function of membrane-bound ion channels, including transporters (Gualtieri *et al.*, 2021). The hexosamine pathway is elevated in diabetes, leading to increased O-GlcNAcylation of proteins, which disrupts cellular signaling and enhances oxidative stress (Buse, 2006). High amounts of reactive oxygen species, such as singlet oxygen, high reactive oxide anions, hydrogen peroxide, and hydroxyl radicals, initiate a chain reaction of oxidation and peroxidation of lipids that produces end metabolites like 4-hydroxynonenal and malondialdehyde, disrupting membrane fluidity and integrity. These ROS also oxidize thiol groups in proteins, impairing enzymes and structural proteins essential for sperm motility and fertility (Ayala *et al*., 2014).

Diabetes-linked oxidative stress can lead to vascular complications, particularly microvascular issues in various visceral organs, including the testis (Zhang *et al*., 2023). This condition interferes with regional and organ-specific hypoxia, potentially causing germ cell damage in seminiferous tubules, as hypoxia is another contributing factor to organ-specific ROS generation (Clanton, 2007). Sperm cells are surrounded by numerous spiral mitochondria, oriented in a sheath along the body and a significant portion of the tail. The mitochondria of sperm are closely associated with the potential and activities of these germ cells, crucial for maintaining fertility (Park & Pang, 2021). Therefore, diabetes-induced oxidative stress is tied to damage to mitochondrial proteins involved in oxidative phosphorylation, such as cytochrome c oxidase and ATP synthase, which disrupts efficient ATP production specifically in sperm cells (Guo *et al*., 2013). Additionally, oxidative damage to mitochondrial DNA can lead to gene mutations, chromatid exchanges, deletions, and abnormal chromatin packaging and ligation during sperm maturation, further aggravating mitochondrial dysfunction (Thomas *et al*., 2021). Excessive ROS contribute to apoptosis and DNA fragmentation of sperm cells through the formation of 8-oxoguanine from guanine oxidation, a mutagenic lesion that can induce mispairing and result in DNA single strand breaks (SSBs). If SSBs are not effectively resolved through the base excision repair (BER) pathway, they can accumulate and transform into double-strand breaks (Aguiar *et al*., 2013). Furthermore, mitochondrial dysfunction in diabetes prompts the release of cytochrome c into the cytosolic compartment, which is associated with apoptotic protease activating factor-1. This leads to the formation of an apoptosis-linked sub-cellular structure known as the apoptosome, which activates caspase-9 and subsequently the effector caspase, caspase-3. Active caspase-3 can cleave and activate caspase-activated DNase (CAD), directly degrading DNA and inducing fragmentation. Simultaneously, the activation of the p53 pathway promotes the transcription of pro-apoptotic genes such as Bax, which increases the permeability of the mitochondrial outer membrane, facilitating cytochrome c release and amplifying the apoptotic death rate of sperm cells.

Compromised membrane integrity and increased permeability allow excessive influx of calcium ions and trigger the externalization of phosphatidylserine, an early apoptotic signal (Furuta *et al.*, 2021). Elevated intracellular calcium levels can also activate various endonucleases, including CAD and endonuclease G, which migrate to the nucleus and cleave DNA (Widłak, 2000). Reactive oxygen species can modify and inactivate DNA repair proteins such as 8-oxo guanine DNA glycosylase-1 and apurinic/apyrimidinic endodeoxyribonuclease 1, regulatory enzymes in the BER pathway, further impairing the recovery of DNA strand damage caused by oxidation and exacerbating DNA fragmentation (de Souza-Pinto *et al*., 2001). Moreover, oxidative modification of histones and protamines, essential for DNA packaging in sperm, leads to chromatin decondensation and increased susceptibility of DNA to oxidative damage and fragmentation (Zini & Libman, 2006; Pal *et al*., 2024b). This molecular cascade ultimately results in compromised sperm genomic integrity and reduced fertilization potential in diabetes. The mechanism of DNA breakage in sperm cells in diabetes has been confirmed here by the Acridine orange study as well as the Annexin V and Comet studies, which are sensitive methods for detecting sperm cell DNA breakage and apoptosis in relation to our previous investigation by TUNEL (Katubi *et al.*, 2019; Pal *et al*., 2024b). Annexin V staining enables early detection of sperm apoptosis, both before and immediately after ejaculation, serving as a unique biomarker for assessing sperm fertility status in ART (Oosterhuis *et al.*, 2000). Such assessments offer a more precise evaluation compared to our previous investigation (Pal *et al.*, 2024b), providing critical information about semen quality and its suitability for processing in assisted reproductive technologies to enhance the likelihood of a successful pregnancy outcome. The major phytocompounds in AEAV were identified via HPLC due to its excellent sensitivity, specificity, efficiency, accuracy, and reliability compared to other chromatographic analyses, especially for mixed samples (Gupta *et al*., 2022). This method was preferred over LC-MS, which was considered in our previous study (Pal *et al.*, 2024b). While LC-MS is powerful, its limitations, including matrix effects, ion suppression, and a diminished linear range (Steinmann & Ganzera, 2011), can be overcome by HPLC, which offers higher reproducibility and resistance to interferences. The tentative phytocompounds were indicated by matching peak absorption and retention time values from chromatogram fingerprinting literature and libraries, demonstrating strong antioxidant capacity, primarily consisting of flavonoids and phenols (Trabelsi *et al*., 2013; Nguyen *et al*., 2018; Farag *et al*., 2020; Nwaso-Sunday *et al*., 2024). Such DNA breakage in sperm, along with ROS-induced changes in mitochondria, can explain the low motility and viability of sperm cells in diabetes. The curative ability of the phytomolecules present in AEAV, due to their antioxidant properties, has been supported in this study by the DPPH assay, as well as a recoverable effect on DNA fragmentation through the Comet and Annexin V assays, which focus on the rectification of spermiological sensors. The role of flavonoid and phenolic compounds (FPC) in this treatment may be explained by their enhancement of ATP production and oxidative phosphorylation while mitigating oxidative stress. These bioactive compounds function as potent antioxidants, scavenging ROS and reducing oxidative damage to mitochondrial membrane proteins critical for energy production. By stabilizing the membrane potential of mitochondria, FPC prevents the leakage of electrons from the ETC, thus minimizing the formation of superoxide anions and other ROS. This protection of ETC integrity ensures efficient oxidative phosphorylation coupling, where graded proton density is maintained along the surfaces of the internal mitochondrial membrane, driving ATP synthesis through ATP synthase (Günther *et al*., 2023). Collectively, these actions maintain the efficiency of mitochondrial ATP production, ensuring that sperm cells have the necessary energy for motility and other essential functions while protecting against oxidative damage. Though there are some contradictory results concerning *Aloe vera (L.)* on male germ cells, where it showed toxic effects on spermiological sensors (Olugbenga *et al.*, 2011), others have reported the curative effect of AEAV on testicular toxicity (Behmanesh *et al.*, 2018; Pal *et al*., 2024a). This contradiction may be due to variations in the applied dose. The present *ex vivo* study, through biochemical and genomic integrity assessments, confirms the antioxidant activity of AEAV at 10-40 mg/10 mL *ex vivo* media, which corresponds to a dose of 10-40 mg/100 g of somatic weight as seen in *in-vivo* studies considering a 10 mL blood volume for such a rat age group. This dose-specific antioxidant activity of the extract aids not only in the recovery from diabetes-induced spermiological damage but also ameliorates oxidative injuries in the testis, liver, and kidney developed due to diabetes. This is a unique aspect of the study, increasing the potential for raising herbal spermiological curators for addressing male infertility challenges, especially in *in-vitro* clinics for managing infertile cases through artificial insemination, including intrauterine insemination and in-vitro blastocyst transfer cases. This also opens another possibility for enhancing sperm activity through exposure to this extract in relation to *ex vivo* fertilization-blastocyst transfer utilized in Technology Assisted Reproduction for cases of male infertility management, where sperm fertility is compromised due to motility, viability, and other related dysfunctions. No toxicity was noted from the AEAV extract on metabolic and accessory reproductive organs concerning acid and alkaline phosphatase, as well as ALT and AST activities, which are important sensors for these purposes (Rahman *et al.*, 2000; Neuman, 2019). The increased activities of prostatic acid and alkaline phosphatases associated with diabetes may be due to diabetes-induced prostatic inflammation (Dokuyucu *et al.*, 2016). Additionally, high prostatic acid phosphatase activities in semen are linked to low sperm motility and fertility (Alkhalaf *et al.*, 2020). Extract treatment has resulted in both phosphatase activities decreasing in the prostate, highlighting its corrective effect on prostatic inflammation as well as its impact on sperm sensors-another dimension of this study compared to our previous investigation in this ar*ea* (Pal *et al*., 2024a; 2024b). The optimum effective dose at the threshold level was 20 mg/10 mL of *ex vivo* media. The effect of other doses may be explained through a phytomolecule-spare receptor study (Homer & Nielsen, 1987; Neubig *et al*., 2003). A high dose of AEAV may desensitize the receptor, while a low dose may cause hypo-saturation kinetics of receptor-phytomolecule-ligand formation.

## CONCLUSIONS

The results highlighted that diabetes causes sperm damage through oxidative stress, which can be reversed by the phytomolecules of AEAV, exerting a direct curative effect on sperm cells affected by oxidative damage associated with diabetes. Due to its antioxidant activity in a dose-specific manner, sperm viability in diabetic state can be preserved. The ex vivo spermiological study aims to address male infertility in chronic diabetes using this extract in both rat and human models, opening the door for further studies employing in vivo models in rats to elucidate the mechanisms of action of the related phytomolecules, ultimately offering insights for drug development in this context. The extract may be utilized for sperm processing to boost the fertility potential of sperm used in ART.
